# Broadening risk profile in familial colorectal cancer type X; increased risk for five cancer types in the national Danish cohort

**DOI:** 10.1186/s12885-020-06859-5

**Published:** 2020-04-22

**Authors:** Christina Therkildsen, Maria Rasmussen, Lars Smith-Hansen, Thomas Kallemose, Lars Joachim Lindberg, Mef Nilbert

**Affiliations:** 1 HNPCC Register, Clinical Research Centre, Copenhagen University Hospital, Kettegård Allé 30, 2650 Hvidovre, Denmark; 2 Digestive Disease Center, Bispebjerg Hospital, University of Copenhagen, Copenhagen, Denmark; 3 Institute of Clinical Sciences, Division of Oncology and Pathology, Lund University, Lund, Sweden; 4 Danish Cancer Society Research Center, the Danish Cancer Society, Copenhagen, Denmark

**Keywords:** Tumour spectrum, Hereditary cancer, Cancer syndrome, Mismatch repair proficient, Amsterdam I criteria

## Abstract

**Background:**

Familial colorectal cancer type X (FCCTX) is a phenotypically defined subset of hereditary colorectal cancer with unknown and potentially heterogeneous genetic aetiology. FCCTX has been characterized as a colorectal cancer-specific syndrome, which we herein challenge by estimating the risk for extra-colorectal cancer in the Danish FCCTX cohort.

**Methods:**

Through the national hereditary non-polyposis colorectal cancer (HNPCC) register, 213 families fulfilling the Amsterdam I criteria and showing retained mismatch repair (MMR) function were identified. In here, sex and age-specific incidence rate ratios (IRR) were calculated for 30 extra-colorectal cancer types in comparison with the general Danish population.

**Results:**

In total, 494 extra-colorectal cancers developed with significantly increased risks for cancers of the urinary tract, breast, stomach, pancreas, and eye tumours. The age groups at increased risks were 30–49 years for gastric cancer, 30–69 years for female breast cancer, 50–69 years for ocular melanoma and above age 70 for pancreatic cancer and urothelial cancer.

**Conclusions:**

Danish FCCTX families show an increased risk of several extra-colorectal cancer types. This observation may indicate unidentified disease-predisposing genetic variants in this phenotypically defined subset of hereditary colorectal cancer and calls for awareness during genetic counselling and follow-up.

## Background

Heredity is estimated to explain ~ 20% of the colorectal cancer diagnoses and covers a complex genetic landscape [35]. Though several rare high-risk alleles have been identified, a large fraction of families with seemingly inherited colorectal cancer diagnoses remains genetically undefined. In here families, who meet the Amsterdam I criteria for Lynch syndrome, but with no signs of mismatch repair (MMR) deficiency, i.e. a mismatch-repair stable phenotype and/or retained MMR protein expression, are referred to as familial colorectal cancer type X (FCCTX) [21, 38]. This subset constitutes 40% of the families that fulfil the Amsterdam I criteria and belong to the hereditary non-polyposis subgroup of hereditary cancer [34]. The genetic aetiology of FCCTX is most likely heterogenous and may include rare pathogenic germline variants in e.g. heterozygous *MUTYH*, *CHEK2*, *BRCA2*, *POLE*, *POLD1*, *SEMA4A*, *BMPR1A*, *RPS20* or *OGG1* or modifying single nucleotide polymorphisms in *SEMA4A, EXO1, TGFBR1,* or *NUDT1* [1, 5, 9, 10, 12, 24, 25, 29, 40].

Besides the MMR proficient molecular phenotype, FCCTX-associated colorectal cancers have been distinguished from the genetically defined cancer syndrome, Lynch syndrome, by a predilection for tumour development in the distal colon and the rectum, a high adenoma/carcinoma rate and a lower risk of synchronous and metachronous colorectal cancer [6, 19, 22, 30]. The risk of colorectal cancer is lower than in Lynch syndrome with a relative risk (RR) of 0.5, but higher than the general population with a standardized incidence ratio of 2.3 [2, 21]. Current literature suggests that FCCTX is a colorectal cancer-only syndrome, which provide the basis for current recommendations of surveillance with regular colonoscopy starting 5–10 years prior to the youngest case in the family in families classified as FCCTX [2, 21, 22, 36].

We challenged this notion through risk assessment of 30 different extra-colorectal cancer types in the Danish FCCTX cohort compared to the Danish general population and to the national Lynch syndrome cohort and found increased risk of five extra-colorectal cancer types, i.e. urothelial cancer, female breast cancer, gastric cancer, pancreatic cancer, and ocular melanoma.

## Methods

The national Danish hereditary non-polyposis colorectal cancer (HNPCC) register contains ~ 6000 families with suspected or verified hereditary colorectal cancer reported to the register by genetic counsellors, surgeons, pathologists, and genetic diagnostic laboratories. Families have been included based on a suspicious family history of colorectal cancer, fulfilment of the Amsterdam I or II criteria [38], or identification of disease-predisposing variants in genes linked to hereditary colorectal cancer. Based on family history, the register subclassifies families according to genotypic and phenotypic subsets [20]. The Danish HNPCC register identifies all family members in the Danish Civil Registration System, regardless of cancer history, based on data collected from clinical files and health care registers.

### Patient selection

Families classified as FCCTX (*n* = 213) were eligible for the study. FCCTX was defined as fulfilment of the Amsterdam I criteria with no signs of MMR deficiency. The Amsterdam I criteria are defined as at least three relatives with histologically verified colorectal cancer in two generations with one individual being a first-degree relative of the other two and at least one individual diagnosed below the age of 50 [37]. MMR proficiency was characterized by retained MMR protein expression and/or a microsatellite stable phenotype and/or a gene test showing no MMR mutations in at least one of the three colorectal cancer patients included in the Amsterdam I triad.

A maximum of one tumour with MLH1/PMS2 protein loss in the family was accepted if this was in conjunction with a *BRAF* mutation and/or *MLH1* promotor hypermethylation (*N* = 4) or normal MMR expression was found in ≥1 tumour from a family member (*N* = 16). Loss of MSH2/MSH6 protein expression was not allowed, while loss of MSH6 only was observed in two cases and allowed motivated by normal genetic test result in the same individual (N = 1) or normal MMR protein expression in another tumour in the same family (N = 1). Variants of unknown significance were included only when normal MMR protein expression was verified in a tumour from the same patient (*N* = 2). Of the 252 Amsterdam I positive families reviewed, 213 fulfilled the criteria for MMR proficiency. Individuals affected with colorectal cancer and their first- and second-degree relatives were eligible for the study.

### Data processing

Data on primary extra-colorectal cancer diagnoses were obtained from the population-based Danish Cancer Registry. This registry has close to complete coverage based on mandatory double reporting from pathologists and clinicians [31, 32]. Benign tumours, carcinoma in situ/dysplasia, and basal cell carcinomas of the skin were excluded. Patients with more than one primary cancer in different organs were allowed to contribute to the tissue-specific risk estimates, while synchronous/metachronous cancer in the same organ or in the same side of paired organs were not allowed. Data on vital status were obtained from the Danish Civil Registration System.

To determine the risk relative to the general population, we used a population-based cohort obtained from the Nordcan database [8]. This cohort contains data on age-specific cancer events and person years at-risk in the Danish background population during the time period from January 1st, 1978 to December 31st, 2013 with stratification for year of diagnosis, sex, age, and disease. The Nordcan database classifies malignancies into 36 groups. The FCCTX-associated cancers could be matched to 30 of these after exclusion of cancer in the colon, rectum and anal canal, unspecified cancers, specified cancer (grouped by Nordcan), and 2 rare specified malignancies without cases in the FCCTX cohort. All cancers and person years at-risk identified in the Danish FCCTX cohort and the previously published Danish Lynch syndrome cohort were removed from the Nordcan data set [33]. To correct for potential ascertainment bias, we performed a subgroup analysis in a cohort surveilled for colorectal cancer, reflecting prospective data, with inclusion of cancers diagnosed following the first colonoscopic surveillance session in the family and exclusion of diagnoses and person years at-risk prior to this date. The study was granted acceptance from the Danish Data Protection Agency. According to Danish regulations, registry studies are not subject to ethical review.

### Statistical analyses

Person years at-risk and cancer events in the FCCTX cohort and in the population-based Nordcan cohort were stratified and aggregated into 4 age groups (0–29 years, 30–49 years, 50–69 years and 70 years or above) using the %STRATIFY SAS macro, which removes individuals from the at-risk group if cancer is diagnosed within the study period, and SAS software, version 9.4 (SAS Institute Inc., Cary, NC, USA) [28]. Person years at-risk were determined as the period from date of birth or start of study period (January 1st, 1978), whichever came last, to date of diagnosis of any type of cancer, date of death or end of study period (December 31st, 2013), whichever came first.

Stratified and aggregated data were transferred into R 3.2.3 (R Core Team, 2019, *A Language and Environment for Statistical Computing*. Vienna, Austria: R Foundation for Statistical Computing: URL https://www.R-project.org/). Incidence rates (IRs) were calculated as the number of events divided by person years at-risk in each age group. Incidence rate ratios (IRRs) were calculated as the ratio between the IRs in the FCCTX cohort and the population-based cohort. Since FCCTX is part of the HNPCC subgroup of hereditary colorectal cancer and are classified according to the same clinical criteria, i.e. the Amsterdam criteria, we also estimated IRs relative to the previous IRs published in the Danish Lynch syndrome cohort [33]. Confidence intervals (95% CI) and *p* values were calculated using the exact conditional Poisson test. All p values were two-sided and significance levels were adjusted for multiple testing using Bonferroni correction for estimation in the 4 age groups (i.e. significance was reached when *p* < 0.0125).

## Results

The FCCTX cohort comprised 213 families, including 646 individuals with a colorectal cancer, 1982 first-degree relatives and 1044 second-degree relatives. These individuals contributed with 110,767 person-years at-risk and during this time 966 individuals developed 1078 cancers, including 493 extra-colorectal cancers and 585 colorectal cancers (Supplementary Table 1). The most prominent cancer types observed were breast cancer (*N* = 103), prostate cancer (*N* = 51), urothelial cancer (*N* = 45), and lung cancer (*N* = 40). Prospective analyses were based on 223 individuals in surveillance from 109 families, who contributed with 903,448 person years at-risk. In this subgroup that considered only cancers that developed after the initiation of surveillance in the family, 160 cancers, including 26 breast cancers, 21 urothelial cancers and 19 prostate cancers, were diagnosed (Supplementary Table 1).

**Fig. 1:**
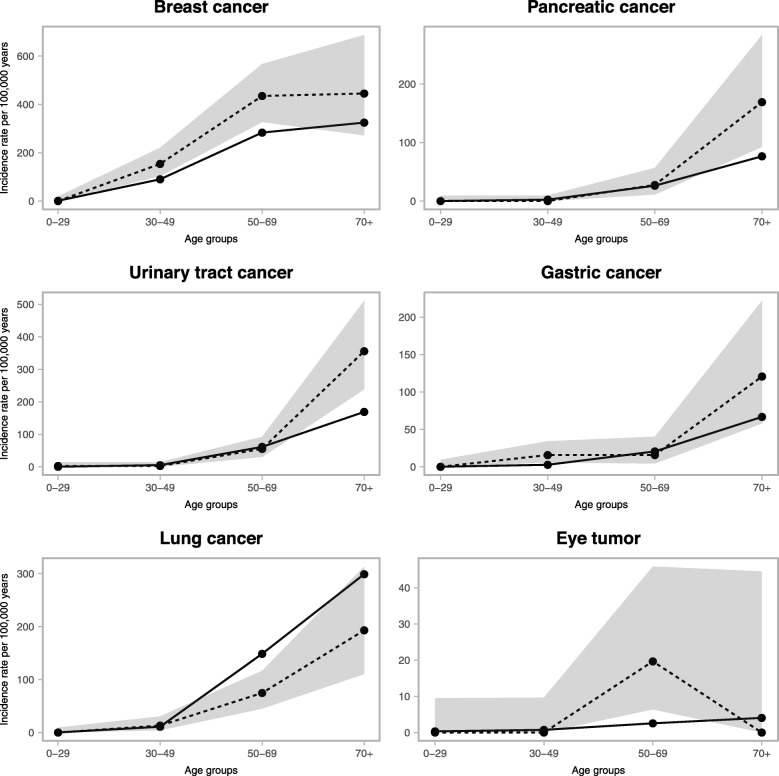
Age-dependent incidence rates and 95% confidence intervals for five cancer types with significantly increased incidence rate ratios in at least one age group in FCCTX (dotted lines) compared to the general population (solid lines)

Compared to a population-based cohort, the FCCTX cohort revealed significantly increased risks for five cancer types, i.e. breast cancer, urothelial cancer, pancreatic cancer, gastric cancer, and ocular melanoma with variable peak incidence ages identified in the different tumour types (Fig. 1, Table 1, Supplementary Table 2). Significantly increased IRRs were observed for breast cancer in the age groups from age 30 until 69 years (IRR for age 30–49: 1.71, 95% CI 1.02–2.68, *p* = 0.0070 and IRR for age 50–69: 1.54, 95% CI 1.06–2.14, *p* = 0.0030), while gastric cancer showed an IRR of 5.87 (95% CI 1.57–15.02, *p* = 0.0007) for the age group 30–49 years. Urothelial cancer and pancreatic cancer developed at significantly increased IRRs of 2.10 (95% CI 1.26–3.30, *p* = 0.0003) and 2.21 (95% CI 1.12–4.38, *p* = 0.0023), respectively, in the oldest age cohort, above age 70. In total, five eye tumours developed in age group 50–69 years, all of which were classified as ocular melanomas, giving an IRR of 7.73 (95% CI 1.76–21.45, *p* = 0.0006) (Fig. 1, Table 1).

**Table 1:** Age-dependent incidence rate ratios of different cancer types comparing the entire FCCTX cohort to the age and sex-matched population-based cohorts

		FCCTX vs. Population-based cohort			
Cancer	Age groups	IRR	95% CI lower	95% CI Upper	***P*** values
**Breast cancer (*n*** **= 103)**	0–29	0.00	0.00	21.39	1.0000
	30–49	1.71	1.02	2.68	0.0070*
	50–69	1.54	1.06	2.14	0.0030*
	70+	1.37	0.72	2.34	0.1495
**Urothelial cancer (*n*** **= 45)**	0–29	10.01	0.06	73.02	0.0955
	30–49	0.50	0.00	3.62	1.0000
	50–69	0.90	0.41	1.70	0.7999
	70+	2.10	1.26	3.30	0.0003*
**Lung cancer (*n*** **= 40)**	0–29	0.00	0.00	81.72	1.0000
	30–49	1.20	0.27	3.32	0.6186
	50–69	0.50	0.26	0.87	0.0011*
	70+	0.65	0.31	1.17	0.0864
**Pancreatic cancer (*n*** **= 21)**	0–29	0.00	0.00	348.69	1.0000
	30–49	0.00	0.00	5.75	1.0000
	50–69	1.05	0.32	2.51	0.8450
	70+	2.21	1.12	4.38	0.0023*
**Gastric cancer (*n*** **= 20)**	0–29	0.00	0.00	136.20	1.0000
	30–49	5.87	1.57	15.02	0.0007*
	50–69	0.77	0.14	2.35	0.8254
	70+	1.81	0.70	3.80	0.0811
**Eye tumours (*n*** **= 5)**	0–29	0.00	0.00	40.95	1.0000
	30–49	0.00	0.00	18.09	1.0000
	50–69	7.73	1.76	21.45	0.0006*
	70+	0.00	0.00	15.20	1.0000

*Significant *p* values following Bonferoni correction

The increased risks of extra-colorectal cancers applied to all FCCTX individuals with similar risk levels in first-degree and second-degree relatives compared to the relatives affected with colorectal cancer (Supplementary Figure 1). In the prospective analysis, significantly increased risks applied to urothelial cancer and ocular melanoma with an IRR of 2.94 (95% CI 1.34–5.54, *p* = 0.0004) above age 70 for urothelial cancers and an IRR of 13.81 (95% CI 1.68–49.32, *p* = 0.0015) for ocular melanomas (Table 2).

**Table 2:** Age-dependent incidence rate ratios comparing the surveilled FCCTX cohort and the population-based cohorts

		Surveilled FCCTX vs. Population-based cohort			
Cancer	Age groups	IRR	95% CI lower	95% CI Upper	***P*** values
**Urothelial cancer (*n*** **= 21)**	0–29	0.00	0.00	301.96	1.0000
	30–49	0.00	0.00	9.19	1.0000
	50–69	1.34	0.41	3.21	0.3786
	70+	2.94	1.34	5.54	0.0004*
**Eye tumours (*n*** **= 3)**	0–29	0.00	0.00	240.15	1.0000
	30–49	0.00	0.00	64.93	1.0000
	50–69	13.81	1.68	49.32	0.0015*
	70+	0.00	0.00	44.19	1.0000

*Significant *p* values following Bonferoni correction

At least one individual with breast cancer was observed in 85 FCCTX families. If the criteria for genetic testing for hereditary breast and ovarian cancer were considered, 35 of these families fulfilled at least one of the criteria previously described [41]. Likewise, gastric cancer was found in 18 FCCTX families, of which two families fulfilled the clinical criteria for genetic testing previously published [4, 26]. As we did not have genetic data on variants in the *BRCA1*/*BRCA2* genes predisposing for hereditary breast and ovarian cancer or the *CDH1* gene predisposing for hereditary diffuse gastric cancer, we excluded these families from the FCCTX cohort. The sensitivity analysis decreased the IRRs to non-significant levels for breast cancer, gastric, pancreatic and urothelial cancer, except for breast cancer age 30–49 where the IRR significantly decreased to 0.19 (95% CI: 0.02–0.67, *p* = 0.0002), from a previous increased IRR. Eye tumours remained significantly increased with similar IRR (Table 3).

**Table 3:** Age-dependent incidence rate ratios of specific cancer types in the FCCTX cohort without the 37 families that fulfilled the criteria for genetic testing for hereditary breast and ovarian cancer or hereditary gastric cancer conpared to the age and sex-matched population-based cohort

		FCCTX subset vs. Population-based cohort			
Cancer	Age groups	IRR	95% CI lower	95% CI Upper	***P*** values
**Breast cancer (*n*** **= 36)**	0–29	0.00	0.00	23.91	1.0000
	30–49	0.19	0.02	0.67	0.0002*
	50–69	0.80	0.46	1.26	0.2639
	70+	0.56	0.19	1.27	0.1104
**Urothelial cancer (*n*** **= 33)**	0–29	10.10	0.06	73.70	0.0947
	30–49	0.51	0.00	3.65	1.0000
	50–69	0.65	0.25	1.36	0.2011
	70+	1.53	0.83	2.59	0.0568
**Lung cancer (*n*** **= 25)**	0–29	0.00	0.00	82.68	1.0000
	30–49	0.97	0.17	2.97	1.0000
	50–69	0.35	0.15	0.67	< 0.0001*
	70+	0.32	0.11	0.74	0.0002*
**Pancreatic cancer (*n*** **= 13)**	0–29	0.00	0.00	351.13	1.0000
	30–49	0.00	0.00	5.78	1.0000
	50–69	0.75	0.17	2.08	0.6967
	70+	1.27	0.42	2.89	0.4249
**Gastric cancer (*n*** **= 12)**	0–29	0.00	0.00	137.04	1.0000
	30–49	2.94	0.36	10.51	0.0840
	50–69	0.38	0.02	1.73	0.2642
	70+	1.27	0.39	3.04	0.5161
**Eye tumours (*n*** **= 4)**	0–29	0.00	0.00	40.98	1.0000
	30–49	0.00	0.00	18.10	1.0000
	50–69	6.19	1.11	19.07	0.0044*
	70+	0.00	0.00	15.20	1.0000

*Significant *p* values following Bonferoni correction

Compared to Lynch syndrome, FCCTX families showed significantly lower risks in at least one age group for eight different tumour types, i.e. endometrial cancer, ovarian cancer, urothelial cancer, kidney cancer, gastric cancer, cancer of the small bowel, non-melanoma skin tumours and brain tumours (Table 4). For urothelial cancer and skin cancer this observation was consistent from age 30+. The risk of urothelial cancer in FCCTX showed IRRs of 0.06–0.31 (*p*-values < 0.0009) compared to Lynch syndrome and the risk of skin cancer showed IRRs of 0.11–0.24 (*p* < 0.005) (Table 4). For the other tumour types this difference particularly applied to the peak ages of these cancer types in Lynch syndrome, i.e. age 30–69 years (Table 4). No difference in risks of breast cancer, eye tumours and pancreatic cancer were found between the FCCTX and Lynch syndrome cohorts for all age groups (Supplementary Table 4.).

**Table 4:** Age-dependent incidence rate ratios of different cancer types comparing the entire FCCTX cohort with the Lynch syndrome cohort

		FCCTX vs. Lynch syndrome			
Cancer	Age groups	IRR	95% CI lower	95% CI Upper	***P*** values
**Urothelial cancer**	0–29	###	0.00	11.07	0.2817
	30–49	###	0.00	0.65	0.0009*
	50–69	###	0.05	0.24	< 0.0001*
	70+	###	0.14	0.72	0.0003*
**Non-melanoma skin tumours**	0–29	NA	0.00	Inf	1.0000
	30–49	###	0.01	0.73	0.0012*
	50–69	###	0.06	0.46	< 0.0001*
	70+	###	0.06	0.94	0.0046*
**Brain tumours**	0–29	###	0.05	20.46	1.0000
	30–49	###	0.05	1.04	0.0102*
	50–69	###	0.09	1.31	0.0283
	70+	NA	0.16	Inf	0.5993
**Gastric cancer**	0–29	NA	0.00	Inf	1.0000
	30–49	###	0.12	5.51	0.7383
	50–69	###	0.02	0.55	0.0001*
	70+	###	0.10	2.38	0.1672
**Ovarian cancer**	0–29	NA	0.05	Inf	0.5384
	30–49	###	0.00	0.22	< 0.0001*
	50–69	###	0.16	3.98	0.5594
	70+	NA	0.09	Inf	1.0000
**Endometrial cancer**	0–29	NA	0.00	Inf	1.0000
	30–49	###	0.00	0.04	< 0.0001*
	50–69	###	0.01	0.08	< 0.0001*
	70+	###	0.03	1.92	0.0441
**Kidney cancer**	0–29	NA	0.00	Inf	1.0000
	30–49	###	0.00	156.30	0.5500
	50–69	###	0.05	0.91	0.0038*
	70+	###	0.02	8.29	0.2207
**Small bowel cancer**	0–29	NA	0.00	Inf	1.0000
	30–49	###	0.00	0.66	0.0009*
	50–69	###	0.00	0.43	0.0001*
	70+	###	0.00	4.47	0.0843

*Significant *p* values following Bonferoni correction

## Discussion

Increased awareness of hereditary colorectal cancer and improved access to genetic diagnostics implies that a growing number of families with a phenotype suggesting hereditary cancer with an undefined genotype are identified. FCCTX represents one of these subsets where refined risk estimates are relevant to develop evidence-based surveillance recommendations. Lindor et al., described a standardized incidence ratio for colorectal cancer of 2.3 in a cohort of 71 FCCTX families and did not identify any significantly increased risk of extra-colorectal cancer [21]. Based on this observation, FCCTX is considered a colorectal cancer-only syndrome with surveillance generally recommended to be confined to colonoscopy with 5-year intervals starting 5–10 years prior to the first case in the family. Surveillance programs for colorectal cancer have been optimized with documentation of more efficient detection of precursor lesions and early-stage tumours (RR 0.2–0.3) [14]. Reduced risk of mortality from colorectal cancer and increased life expectancy implies that individuals with FCCTX may be at risk of extra-colorectal tumour types during this increased lifetime. Our data, based on all 213 FCCTX families in the national Danish HNPCC-register, challenges the present view on FCCTX as a colorectal cancer-only syndrome and demonstrate significantly increased incidence rates for five extra-colorectal cancer types with urothelial cancer remaining significant in the colorectal cancer-surveilled cohort (Fig. 1, Table 1, Table 2).

We demonstrate an increased risk of urothelial cancer from age 70 in FCCTX with IRRs of 2.1 in the entire FCCTX cohort and 2.9 in the surveilled subset compared to the risk in an age- and sex matched Danish population (Tables 1 and 2). The risk of urothelial cancer in FCCTX was significantly lower than in Lynch syndrome with IRRs of 0.1–0.3 (Table 4). Except for Lynch syndrome, urothelial cancer has not been linked to hereditary colorectal cancer [11]. One possibility would be undiagnosed Lynch syndrome cases e.g. MMR gene variants that allow for retained MMR function and normal MMR protein expression. Alternatively, a subset of FCCTX could harbour mutations in genes linked to urothelial cancer development, e.g. *FGFR3*, *TP53* or *HRAS* [43]. To this point, 42/45 urothelial cancer in the FCCTX cohort developed in the urinary bladder, which stands in contrast to a predilection for tumours in the upper urinary tract in Lynch syndrome [17]. Unfortunately, it was not possible to discriminate between upper and lower urinary tract cancers in the population-based cohort since the Nordcan database does not differentiate between these sites.

The increased risk of gastric cancer with an IRR of 5.9 in the age group 30–49 years could potentially signify a genetic subset that confers heredity for this cancer type. About 10–20% of gastric cancer is caused by heredity with confirmed causes in 1–3%, predominantly linked to the hereditary diffuse gastric cancer caused by pathogenic variants in the *CDH1* gene [27]. Only two of the 18 FCCTX families in our cohort in which gastric cancer developed fulfilled the criteria currently applied for genetic diagnostics due to early-onset cases in the families [4, 26]. When we excluded these families from the FCCTX cohort the risk was, as expected, reduced and was not significant. The co-occurrence of gastric and colorectal cancer in Amsterdam I positive families calls for further studies but may be explained by polygenetic defects resulting in a severe cancer phenotype in some FCCTX families. Addition of *CDH1* in the genetic testing of FCCTX families might genetically classify a small fraction of the FCCTX families.

The increased risks of breast cancer with IRRs 1.5–1.7 in the age group 30–69 years and pancreatic cancer with an IRR of 2.2 after age 70 support the suggestion of disease-predisposing variants in *BRCA2,* causing the observed malignancies in a small subset of FCCTX families [10]. We also identified an IRR of 10.2 for early-onset ovarian cancer. Various guidelines exist for referring individuals to genetic diagnostics in hereditary breast and ovarian cancer [41]. In addition to fulfilling the Amsterdam I criteria, 35 out of the 213 FCCTX families also fulfilled the clinical genetic testing criteria for hereditary breast and ovarian cancer. Exclusion of these families from the FCCTX cohort decreased the risk of breast and pancreatic cancer to a nonsignificant level for the age groups 50–69 and 70+, and the risk for breast cancer flipped to a significantly decreased IRR when comparing to the population-based cohort for the age group 30–49 years (Table 3). Whether the increased risk of breast cancer and colorectal cancer in these families can be explained solely by *BRCA2* germline mutations or by polygenetic defects or environmental factors remains to be elucidated. Though the families included in this study had not been systematically screened for hereditary breast and ovarian cancer, our data support a role for hereditary breast and pancreatic cancer in FCCTX, and application of broader diagnostic genetic panels that also cover the *BRCA2* gene may, based on our data, have a potential to identify disease-predisposing mutations in some FCCTX families.

The five eye tumours identified in the Danish FCCTX cohort were all malignant melanomas. Uveal melanoma is predominantly sporadic but between 2 and 5% have been estimated to be caused by familial or hereditary predisposition. Autosomal dominant inheritance of pathogenic *BAP1* gene variants have been observed in 47% of uveal melanomas, while pathogenic *EIF1AX* variants have been found in 14–20% of the cases [13, 15]. BAP1-associated uveal melanomas are diagnosed in the age of 30–59 years and are associated with cutaneous melanomas and renal cell carcinomas, while *EIF1AX* gene variants are associated with thyroid and ovarian cancer [13, 15]. In our cohort, uveal melanomas presented in the age span from 54 to 69 years and 2 of 5 cases occurred in patients with previous cutaneous melanomas. These data encourage awareness of family history during the genetic counselling and diagnostic testing.

Comparison between the risk of extra-colorectal cancer in the FCCTX cohort with the national Danish Lynch syndrome cohort, revealed differences as well as similarities. Urothelial cancer and skin cancer showed significantly lower risk levels in FCCTX compared to Lynch syndrome with IRRs of 0.06–0.31 and 0.11–0.24, respectively (Table 4). Increased risks and reminiscent risk profiles applied to breast cancer, gastric cancer and pancreatic cancer. These similarities are also supported by other studies on the risk of upper gastrointestinal cancer in Lynch syndrome [7, 18, 39]. Surveillance for cancer of the upper gastrointestinal tract is not recommended in FCCTX, but the increased risk observed may suggest awareness with consideration of *Helicobacter Pyroli* screening and eradication in FCCTX similarly to the recommendations in Lynch syndrome [16, 38, 39].

Studies have shown that compared to Lynch syndrome, FCCTX confers a lower risk for colorectal cancer (11–20% vs 58–75%), a higher age at onset (60 vs 45 years), a different predominant tumour locations (distal vs proximal) and a worse prognosis [3, 21, 23, 42]. In FCCTX, colonoscopic screening is generally recommended with 5-year intervals starting 5–10 years before the earliest colorectal cancer diagnosis in the family, though surveillance patterns are likely more variable in FCCTX than in Lynch syndrome. The recent demonstration of excess cancer-related deaths in FCCTX compared to Lynch syndrome and short intervals to second primary colorectal cancer suggests that clinical management in FCCTX needs to be optimized [3].

## Conclusions

Our observation of increased risks with distinct and variable incidence patterns in relation to age for five extra-colorectal cancer types in FCCTX needs validation but challenges the present view of FCCTX as a colorectal cancer-only syndrome. The consistently increased risk of urothelial cancer motivates further investigation to obtain more detailed insights into risk profiles and tumour types with the aim to identify possible disease-predisposing genes. The demonstration of increased risks for breast cancer and pancreatic cancer could suggest that genetic variants in *BRC*A2 may explain some FCCTX families. The FCCTX cohort may be a suitable target for application of broader panels during genetic diagnostics. Further characterization and subdivision of FCCTX are needed to define discriminatory features, provide more robust risk estimates and recommend relevant and cost-effective surveillance to individuals at increased risk.

## Supplementary information

**Additional file 1 Table S1** Extracolonic cancers in the entire and the surveilled FCCTX cohort

**Additional file 2 Table S2** Age-dependent incidence rates of different cancer types in the entire FCCTX cohort and in the age and sex-matched population-based cohorts.

**Additional file 3 Figure S1** Impact from degree of relatedness in FCCTX families in the 6 extracolorectal cancer types that were significantly different from the population-based cohort. A) Table showing the number of cancers observed and the mean age at onset in individuals affected with colorectal cancer, their first-degree relatives and their second-degree relatives. B) Incidence rates calculated separately in individuals affected by colorectal cancer (red), first-degree relatives (blue) and second-degree relatives (green). No significant differences were observed. Incidence rates and *p* values are available upon request.

**Additional file 4 Table S3** Age-dependent incidence rates of different cancer types in the surveilled FCCTX cohort and in the age and sex-matched population-based cohorts

**Additional file 5 Table S4**. Age-dependent incidence rates for cancer types in the entire FCCTX cohort compared to the Danish Lynch syndrome cohort.

## Abbreviations

FCCTX: Familial colorectal cancer type X
HNPCC: Hereditary non-polyposis colorectal cancer
IR: Incidence rate
IRR: Incidence rate ratio
MMR: Mismatch repair
*MLH1*: MutL homolog 1
*MSH2*: MutS homolog 2
*MSH6*: MutS homolog 6
*PMS2*: PMS1 homolog 2
*BRAF*: B-Raf protooncogene
*MUTYH*: MutY DNA glucosylase
*CHEK2*: Checkpoint kinase 2
*BRCA1*: Breast cancer 1
*BRCA2*: Breast cancer 2
*POLE*: DNA polymerase epsilon
*POLD1*: DNA polymerase delta 1
*SEMA4A*: Semaphorin 4A
*BMPR1A*: Bone morphogenetic protein receptor type 1A
*RPS20*: ribosomal protein S20
*OGG1*: 8-oxoguanine DNA glycosylase
*EXO1*: Exonuclease 1
*TGFBR1*: Transforming growth factor beta receptor 1
*NUDT1*: Nudix hydrolase 1
*CDH1*: Cadherin 1
*FGFR3*: Fibroblast growth factor receptor 3
*TP53*: Tumour protein p53
*HRAS*: H-Ras protooncogene
*BAP1*: BRCA1 associated protein 1
*EIF1AX*: Eukaryotic translation initiation factor 1A X-linked
