# Supplementary material for: Broadening risk profile in familial colorectal cancer type X; increased risk for five cancer types in the national Danish cohort
Source: BMC Cancer. 2020 Apr 22;20:345. doi: 10.1186/s12885-020-06859-5 (PMC7179001; doi:10.1186/s12885-020-06859-5)
Supplement: Supplementary file 1 — Additional file 1 Table S1 Extracolonic cancers in the entire and the surveilled FCCTX cohort [file 12885_2020_6859_MOESM1_ESM.pdf]

Supplementary table 1 Extracolonic cancers in the entire and the surveilled FCCTX cohort

	Entire FCCTX cohort			Surveilled FCCTX cohort		
**Malignancy**	**Cancers**	**Mean age (years)**	**Range (years)**	**Cancers**	**Mean age (years)**	**Range (years)**
Breast cancer	103	58.0	30-87	26	61.6	36-87
Prostate cancer	51	70.7	55-94	19	73.4	58-89
Urothelial cancer	45	69.7	28-89	21	70.0	50-89
Lung cancer	40	65.3	43-89	10	62.5	46-78
Malignant melanoma	25	53.1	23-80	11	51.1	23-80
Non-melanoma skin cancer	22	66.8	24-88	11	69.7	47-88
Brain tumours	22	55.8	0-93	10	61.5	24-93
Pancreatic cancer	21	73.0	61-89	3	76.0	69-81
Gastric cancer	20	63.4	39-85	5	69.2	48-85
Ovarian cancer	17	57.1	26-79	4	58.3	49-66
Lip and oropharyngeal cancer	15	63.1	47-89	5	64.6	47-89
Non-Hodgkin's lymphoma	15	60.2	41-82	5	55.6	41-74
Endometrial cancer	11	65.4	52-82	3	58.0	52-66
Kidney cancer	10	62.7	47-74	2	66.0	58-74
Cervical cancer	9	46.0	32-71	2	50.5	49-52
Leukemia	8	69.0	59-79	1	70.0	-
Esophageal cancer	6	70.5	50-91	1	64.0	-
Soft tissue tumours	6	53.7	36-71	3	52.0	43-61
Testis cancer	6	34.0	27-36	2	34.0	34-34
Biliary tract cancer	5	58.2	41-79	0	-	-
Laryngeal cancer	5	62.0	53-73	2	59.0	57-61
Eye tumours	5	66.0	54-69	3	65.3	61-69
Multiple myeloma	5	80.8	66-91	2	85.0	79-91
Hepatocellular cancer	4	68.8	68-69	2	68.5	68-69
Other specified cancers	4	61.0	61-65	2	62.5	46-78
Small bowel cancer	3	66.0	49-86	1	63.0	-
Vaginal and vulvar cancer	3	59.3	47-73	1	47.0	-
Cancer of the nose/sinuses	2	50.0	29-71	1	71.0	-
Thyroid cancer	2	67.5	62-73	0	-	-
Pleural mesothelioma	1	68.0	-	1	68.0	-
Bone cancer	1	28.0	-	0	-	-
Hodgkin's lymphoma	1	22.0	-	1	22.0	-
Total	493	59.8	0-94	160	61.4	23-93

*Other specified cancers included 4 cancers of the retroperitoneum
