# Supplementary material for: Broadening risk profile in familial colorectal cancer type X; increased risk for five cancer types in the national Danish cohort
Source: BMC Cancer. 2020 Apr 22;20:345. doi: 10.1186/s12885-020-06859-5 (PMC7179001; doi:10.1186/s12885-020-06859-5)
Supplement: Supplementary file 2 — Additional file 2 Table S2 Age-dependent incidence rates of different cancer types in the entire FCCTX cohort and in the age and sex-matched population-based cohorts. [file 12885_2020_6859_MOESM2_ESM.pdf]

Supplementary Table 2 Age-dependent incidence rates of different cancer types in the entire FCCTX cohort and in the age and sex-matched population-based cohorts

		FCCTX per 100000 years			Population-based cohort per 100000 years			FCCTX vs. Population-based cohort			
**Cancer**	**Age groups**	**IR**	**95% CI lower**	**95% CI Upper**	**IR**	**95% CI lower**	**95% CI Upper**	**IRR**	**95% CI lower**	**95% CI Upper**	**P values**
**Breast cancer (n=103)**	0-29	0.00	0.00	19.08	1.23	1.12	1.35	0.00	0.00	21.39	1.0000
	30-49	153.79	102.99	220.86	90.00	88.87	91.15	1.71	1.02	2.68	**0.0070***
	50-69	435.03	326.81	567.62	283.38	281.13	285.64	1.54	1.06	2.14	**0.0030***
	70+	445.01	271.83	687.29	324.72	321.52	327.94	1.37	0.72	2.34	0.1495
**Prostate cancer (n=51)**	0-29	0.00	0.00	18.99	0.01	0.00	0.02	0.00	0.00	22512.58	1.0000
	30-49	0.00	0.00	19.26	1.57	1.43	1.73	0.00	0.00	16.96	1.0000
	50-69	210.97	137.81	309.11	151.90	150.22	153.59	1.39	0.80	2.23	0.1036
	70+	742.25	480.35	1095.71	621.85	616.44	627.30	1.19	0.68	1.93	0.3799
**Urothelial cancer (n=45)**	0-29	2.58	0.07	14.35	0.26	0.22	0.30	10.01	0.06	73.02	0.0955
	30-49	2.62	0.07	14.59	5.19	5.00	5.39	0.50	0.00	3.62	1.0000
	50-69	55.18	30.17	92.59	61.32	60.57	62.07	0.90	0.41	1.70	0.7999
	70+	355.87	238.33	511.09	169.09	167.30	170.89	2.10	1.26	3.30	**0.0003***
**Lung cancer (n=40)**	0-29	0.00	0.00	9.50	0.16	0.14	0.20	0.00	0.00	81.72	1.0000
	30-49	13.09	4.25	30.55	10.92	10.64	11.20	1.20	0.27	3.32	0.6186
	50-69	74.75	45.00	116.73	148.71	147.55	149.88	0.50	0.26	0.87	**0.0011***
	70+	193.14	110.40	313.65	299.16	296.78	301.55	0.65	0.31	1.17	0.0864
	0-29	7.73	1.59	22.59	3.28	3.15	3.42	2.36	0.29	8.40	0.1370
**Malignant melanoma (n=24)**	30-49	23.59	10.79	44.79	19.59	19.22	19.97	1.20	0.43	2.63	0.5786
	50-69	27.59	11.09	56.84	34.72	34.16	35.29	0.79	0.24	1.90	0.7341
	70+	60.78	19.73	141.83	47.71	46.76	48.67	1.27	0.29	3.52	0.6058
**Non-melanoma skin tumors**	0-29	2.58	0.07	14.36	0.25	0.22	0.29	10.17	0.06	74.22	0.0941
**(n=22)**	30-49	5.24	0.63	18.92	2.69	2.56	2.83	1.95	0.11	8.77	0.2747
	50-69	39.39	18.89	72.44	20.79	20.36	21.23	1.89	0.73	3.98	0.0483
	70+	109.83	50.22	208.49	117.75	116.26	119.25	0.93	0.34	2.03	1.0000
**Brain tumours (n=22)**	0-29	7.73	1.59	22.60	5.32	5.16	5.49	1.45	0.18	5.18	0.4677
	30-49	13.10	4.25	30.57	14.80	14.48	15.13	0.89	0.20	2.45	1.0000
	50-69	31.47	13.59	62.01	35.86	35.29	36.44	0.88	0.29	2.00	0.8683
	70+	72.50	26.61	157.80	46.31	45.38	47.26	1.57	0.42	4.00	0.2939
**Pancreatic cancer (n=21)**	0-29	0.00	0.00	9.51	0.04	0.03	0.06	0.00	0.00	348.69	1.0000
	30-49	0.00	0.00	9.66	2.31	2.19	2.45	0.00	0.00	5.75	1.0000
	50-69	27.53	11.07	56.72	26.24	25.76	26.74	1.05	0.32	2.51	0.8450
	70+	169.16	92.48	283.82	76.49	75.29	77.71	2.21	1.12	4.38	**0.0023***
**Gastric cancer (n=20)**	0-29	0.00	0.00	9.50	0.10	0.08	0.12	0.00	0.00	136.20	1.0000
	30-49	15.71	5.76	34.19	2.68	2.54	2.82	5.87	1.57	15.02	**0.0007***
	50-69	15.73	4.29	40.27	20.56	20.13	21.00	0.77	0.14	2.35	0.8254
	70+	120.69	57.88	221.96	66.70	65.58	67.83	1.81	0.70	3.80	0.0811

**Ovarian cancer (n=17)**	0-29	10.31	1.25	37.26	1.01	0.91	1.12	10.20	0.59	46.32	0.0170
	30-49	10.51	1.27	37.95	10.66	10.27	11.06	0.99	0.06	4.44	1.0000
	50-69	69.27	31.67	131.49	47.75	46.83	48.68	1.45	0.52	3.16	0.2280
	70+	84.24	22.95	215.70	63.23	61.82	64.66	1.33	0.24	4.09	0.5520
**Non-Hodgkin's lymphoma (n=15)**	0-29	0.00	0.00	9.50	1.44	1.36	1.53	0.00	0.00	9.10	1.0000
	30-49	7.86	1.62	22.96	6.44	6.23	6.66	1.22	0.15	4.34	0.7415
	50-69	31.52	13.61	62.10	25.97	25.49	26.46	1.21	0.41	2.76	0.5543
	70+	48.36	13.18	123.81	56.35	55.32	57.39	0.86	0.15	2.64	1.0000
**Head and neck cancer (n=15)**	0-29	0.00	0.00	9.50	0.30	0.26	0.34	0.00	0.00	43.74	1.0000
	30-49	5.24	0.63	18.91	5.28	5.09	5.47	0.99	0.06	4.47	1.0000
	50-69	35.43	16.20	67.25	29.77	29.25	30.30	1.19	0.43	2.59	0.5812
	70+	48.40	13.19	123.92	36.17	35.35	37.01	1.34	0.24	4.11	0.5511
**Endometrial cancer (n=11)**	0-29	0.00	0.00	19.02	0.15	0.12	0.20	0.00	0.00	178.91	1.0000
	30-49	0.00	0.00	19.37	5.35	5.08	5.64	0.00	0.00	4.99	0.6321
	50-69	53.71	21.59	110.65	62.20	61.15	63.27	0.86	0.26	2.07	0.8605
	70+	83.83	22.84	214.63	78.96	77.39	80.56	1.06	0.19	3.26	0.7940
**Kidney cancer (n=10)**	0-29	0.00	0.00	9.50	0.48	0.43	0.53	0.00	0.00	27.39	1.0000
	30-49	2.62	0.07	14.59	3.24	3.09	3.40	0.85	0.01	5.80	1.0000
	50-69	23.59	8.66	51.35	21.65	21.21	22.10	1.11	0.29	2.78	0.8286
	70+	36.18	7.46	105.75	43.13	42.23	44.05	0.86	0.10	2.99	1.0000
**Cervical cancer (n=9)**	0-29	0.00	0.00	19.02	3.25	3.07	3.45	0.00	0.00	8.06	1.0000
	30-49	26.34	8.55	61.47	25.75	25.15	26.37	1.02	0.23	2.83	0.8207
	50-69	23.15	4.77	67.64	26.48	25.79	27.18	0.87	0.11	3.11	1.0000
	70+	21.01	0.53	117.05	28.50	27.56	29.47	0.74	0.00	5.29	1.0000
**Leukemia (n=7)**	0-29	0.00	0.00	9.50	3.29	3.16	3.42	0.00	0.00	3.98	0.6436
	30-49	0.00	0.00	9.66	4.16	3.99	4.33	0.00	0.00	3.20	0.4177
	50-69	11.79	2.43	34.45	21.29	20.85	21.74	0.55	0.07	1.97	0.3915
	70+	48.32	13.16	123.71	62.52	61.44	63.62	0.77	0.14	2.37	0.8250
**Esophageal cancer (n=6)**	0-29	0.00	0.00	9.50	0.01	0.00	0.02	0.00	0.00	2014.24	1.0000
	30-49	0.00	0.00	9.66	1.10	1.01	1.19	0.00	0.00	12.14	1.0000
	50-69	15.72	4.28	40.25	13.19	12.84	13.54	1.19	0.21	3.66	0.5838
	70+	24.17	2.93	87.33	27.20	26.49	27.93	0.89	0.05	4.00	1.0000
**Connecting tissue tumours (n=6)**	0-29	0.00	0.00	9.50	0.82	0.75	0.89	0.00	0.00	16.07	1.0000
	30-49	5.24	0.63	18.92	2.00	1.88	2.12	2.62	0.15	11.84	0.1779
	50-69	11.80	2.43	34.47	4.83	4.62	5.04	2.44	0.30	8.72	0.1267
	70+	12.06	0.31	67.18	9.84	9.41	10.28	1.23	0.01	8.81	0.5578
**Testis cancer (n=6)**	0-29	5.15	0.13	28.69	7.86	7.58	8.15	0.65	0.00	4.71	1.0000
	30-49	26.21	8.51	61.17	19.36	18.85	19.89	1.35	0.31	3.74	0.4283
	50-69	0.00	0.00	29.77	5.48	5.17	5.81	0.00	0.00	7.48	1.0000
	70+	0.00	0.00	104.73	2.80	2.44	3.18	0.00	0.00	52.13	1.0000

**Biliary tract cancer (n=5)**	0-29	0.00	0.00	9.50	0.01	0.00	0.02	0.00	0.00	2014.24	1.0000
	30-49	5.24	0.63	18.91	0.52	0.46	0.58	10.16	0.59	46.26	0.0172
	50-69	7.86	0.95	28.39	6.01	5.78	6.25	1.31	0.08	5.89	0.6686
	70+	12.06	0.31	67.18	22.92	22.26	23.58	0.53	0.00	3.78	1.0000
**Eye tumours (n=5)**	0-29	0.00	0.00	9.50	0.32	0.28	0.37	0.00	0.00	40.95	1.0000
	30-49	0.00	0.00	9.66	0.74	0.67	0.82	0.00	0.00	18.09	1.0000
	50-69	19.66	6.38	45.88	2.54	2.39	2.70	7.73	1.76	21.45	**0.0006***
	70+	0.00	0.00	44.53	4.04	3.77	4.33	0.00	0.00	15.20	1.0000
**Multiple myeloma (n=5)**	0-29	0.00	0.00	9.50	0.01	0.00	0.02	0.00	0.00	3328.36	1.0000
	30-49	0.00	0.00	9.66	1.01	0.93	1.10	0.00	0.00	13.16	1.0000
	50-69	3.93	0.10	21.89	10.20	9.90	10.51	0.39	0.00	2.77	0.5312
	70+	48.28	13.16	123.62	26.70	25.99	27.42	1.81	0.32	5.56	0.1831
**Laryngel cancer (n=5)**	0-29	0.00	0.00	9.50	0.02	0.01	0.03	0.00	0.00	761.64	1.0000
	30-49	0.00	0.00	9.66	1.39	1.30	1.50	0.00	0.00	9.57	1.0000
	50-69	15.73	4.28	40.27	12.63	12.30	12.98	1.24	0.22	3.82	0.5699
	70+	12.08	0.31	67.32	14.47	13.95	15.01	0.83	0.01	6.00	1.0000
**Hepatocellular cancer (n=4)**	0-29	0.00	0.00	9.50	0.17	0.14	0.20	0.00	0.00	79.03	1.0000
	30-49	0.00	0.00	9.66	0.98	0.90	1.07	0.00	0.00	13.61	1.0000
	50-69	15.72	4.28	40.24	10.13	9.83	10.44	1.55	0.28	4.77	0.3352
	70+	0.00	0.00	44.48	25.96	25.26	26.67	0.00	0.00	2.36	0.2874
**Small bowel cancer (n=3)**	0-29	0.00	0.00	9.50	0.03	0.02	0.05	0.00	0.00	467.06	1.0000
	30-49	2.62	0.07	14.59	0.40	0.35	0.46	6.50	0.04	47.31	0.1432
	50-69	3.93	0.10	21.90	2.86	2.70	3.03	1.37	0.01	9.88	0.5173
	70+	12.06	0.31	67.20	6.32	5.98	6.68	1.91	0.01	13.72	0.4081
**Vulva and vaginal cancer (n=3)**	0-29	0.00	0.00	19.02	0.10	0.07	0.14	0.00	0.00	280.20	1.0000
	30-49	5.25	0.13	29.25	1.73	1.58	1.90	3.03	0.02	21.87	0.2818
	50-69	7.67	0.19	42.76	6.69	6.35	7.05	1.15	0.01	8.25	0.5819
	70+	20.96	0.53	116.79	21.85	21.02	22.70	0.96	0.01	6.89	1.0000
**Thyroid cancer (n=2)**	0-29	0.00	0.00	9.50	0.72	0.66	0.78	0.00	0.00	18.24	1.0000
	30-49	0.00	0.00	9.66	3.31	3.16	3.46	0.00	0.00	4.02	0.6427
	50-69	3.93	0.10	21.90	4.10	3.91	4.30	0.96	0.01	6.89	1.0000
	70+	12.07	0.31	67.24	6.37	6.02	6.72	1.90	0.01	13.64	0.4101
**Nose and sinuses (n=2)**	0-29	2.58	0.07	14.35	0.06	0.04	0.08	45.04	0.28	348.07	0.0225
	30-49	0.00	0.00	9.66	0.43	0.38	0.49	0.00	0.00	31.20	1.0000
	50-69	0.00	0.00	14.49	2.39	2.24	2.54	0.00	0.00	8.37	1.0000
	70+	12.07	0.31	67.24	4.76	4.46	5.07	2.54	0.02	18.25	0.3262
**Pleural mesothelioma (n=1)**	0-29	0.00	0.00	9.50	0.01	0.01	0.02	0.00	0.00	1433.01	1.0000
	30-49	0.00	0.00	9.66	0.30	0.26	0.35	0.00	0.00	45.09	1.0000
	50-69	3.93	0.10	21.89	3.47	3.29	3.65	1.13	0.01	8.14	0.5867
	70+	0.00	0.00	44.48	7.17	6.80	7.55	0.00	0.00	8.55	1.0000

**Bone cancer/osteosarcoma (n=1)**	0-29	2.58	0.07	14.35	0.84	0.78	0.91	3.07	0.02	22.11	0.2786
	30-49	0.00	0.00	9.66	0.68	0.61	0.75	0.00	0.00	19.68	1.0000
	50-69	0.00	0.00	14.49	1.02	0.93	1.13	0.00	0.00	19.59	1.0000
	70+	0.00	0.00	44.48	1.71	1.53	1.90	0.00	0.00	36.15	1.0000
**Hodgkin's lymphoma (n=1)**	0-29	2.58	0.07	14.36	2.02	1.92	2.13	1.27	0.01	9.16	0.5440
	30-49	0.00	0.00	9.66	2.52	2.39	2.66	0.00	0.00	5.29	1.0000
	50-69	0.00	0.00	14.49	2.63	2.48	2.79	0.00	0.00	7.60	1.0000
	70+	0.00	0.00	44.48	3.43	3.18	3.70	0.00	0.00	17.90	1.0000

*Signficant p values following Bonferoni correction
