# Supplementary material for: Broadening risk profile in familial colorectal cancer type X; increased risk for five cancer types in the national Danish cohort
Source: BMC Cancer. 2020 Apr 22;20:345. doi: 10.1186/s12885-020-06859-5 (PMC7179001; doi:10.1186/s12885-020-06859-5)
Supplement: Supplementary file 3 — Additional file 3 Figure S1 Impact from degree of relatedness in FCCTX families in the 6 extracolorectal cancer types that were significantly different from the population-based cohort. A) Table showing the number of cancers observed and the mean age at onset in individuals affected with colorectal cancer, their first-degree relatives and their second-degree relatives. B) Incidence rates calculated separately in individuals affected by colorectal cancer (red), first-degree relatives (blue) and second-degree relatives (green). No significant differences were observed. Incidence rates and p values are available upon request. [file 12885_2020_6859_MOESM3_ESM.pdf]

A

| Malignancy | Number in affected* | Mean age (range) | Number in FDR | Mean age (range) | Number in SDR | Mean age (range) |
|---|---|---|---|---|---|---|
| Breast cancer | 19 | 63.9 (45-86) | 70 | 58.0 (30-87) | 15 | 51.7 (31-73) |
| Urothelial cancer | 9 | 71.0 (57-83) | 32 | 70.9 (44-89) | 4 | 57.5 (28-76) |
| Lung cancer | 4 | 71.5 (65-78) | 33 | 65.9 (43-89) | 3 | 50.7 (46-57) |
| Pancreatic cancer | 6 | 73.7 (62-88) | 15 | 72.8 (61-89) | 0 | - |
| Gastric cancer | 5 | 66.0 (48-85) | 13 | 64.0 (39-79) | 2 | 53.0 (44-62) |
| Eye tumours | 1 | 67 (-) | 3 | 61.3 (54-69) | 1 | 66 (-) |

*Affected with colorectal cancer

FDR: first-degree relative to an individual with colorectal cancer

SDR: second-degree relative to an individual with colorectal cancer

B

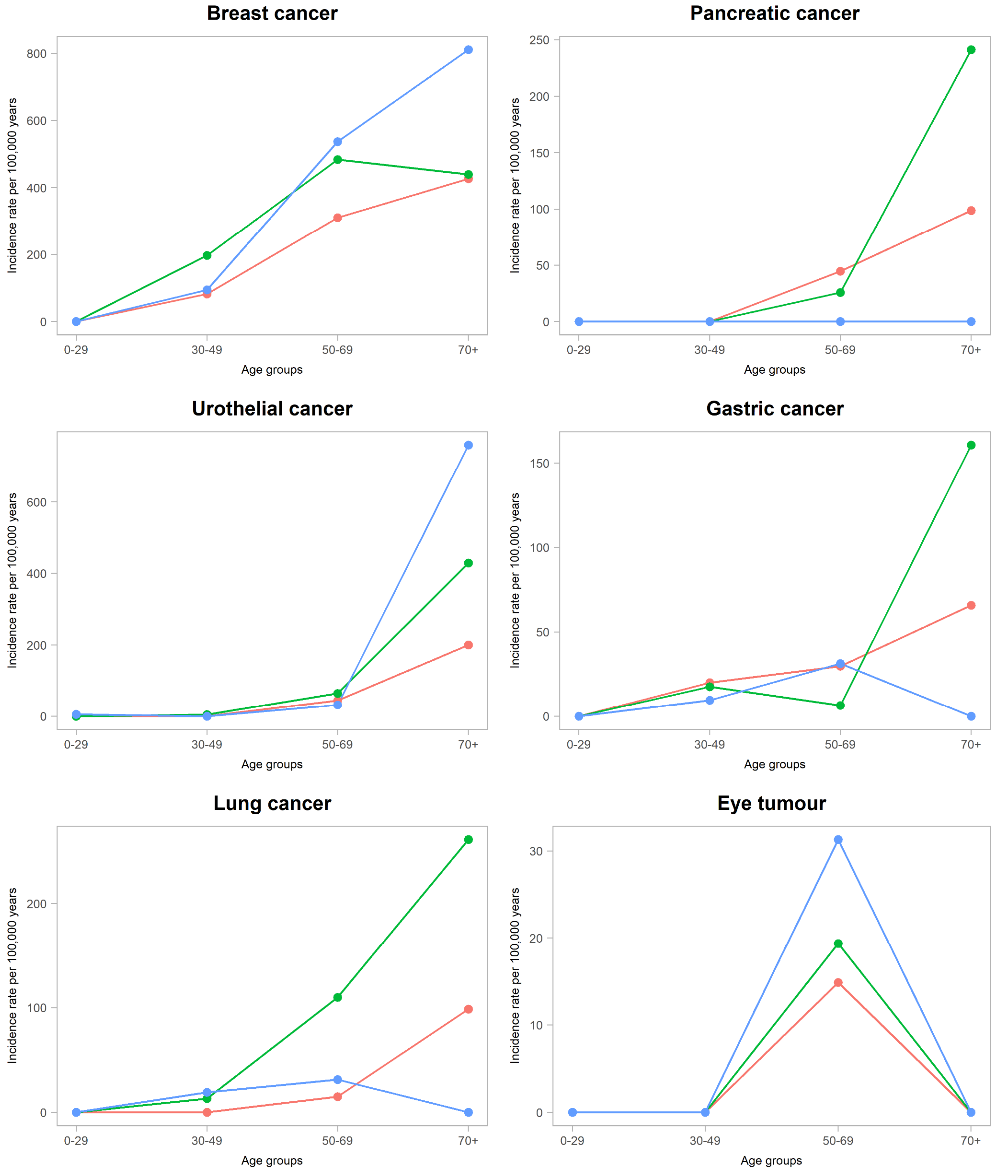


**Supplementary figure 1** Impact from degree of relatedness in FCCTX families in the 6 extra-colorectal cancer types that were significantly different from the population-based cohort. A) Table showing the number of cancers observed and the mean age at onset in individuals affected with colorectal cancer, their first-degree relatives and their second-degree relatives. B) Incidence rates calculated separately in individuals affected by colorectal cancer (red), first-degree relatives (blue) and second-degree relatives (green). No significant differences were observed. Incidence rates and p values are available upon request.
