# Supplementary material for: Broadening risk profile in familial colorectal cancer type X; increased risk for five cancer types in the national Danish cohort
Source: BMC Cancer. 2020 Apr 22;20:345. doi: 10.1186/s12885-020-06859-5 (PMC7179001; doi:10.1186/s12885-020-06859-5)
Supplement: Supplementary file 4 — Additional file 4 Table S3 Age-dependent incidence rates of different cancer types in the surveilled FCCTX cohort and in the age and sex-matched population-based cohorts [file 12885_2020_6859_MOESM4_ESM.pdf]

Supplementary Table 3 Age-dependent incidence rates of different cancer types in the surveilled FCCTX cohort and in the age and sex-matched population-based cohorts

		FCCTX per 100000 years			Population-based cohort per 100000 years			FCCTX vs. Population-based cohort			
**Cancer**	**Age groups**	**IR**	**95% CI lower**	**95% CI Upper**	**IR**	**95% CI lower**	**95% CI Upper**	**IRR**	**95% CI lower**	**95% CI Upper**	**P values**
**Breast cancer (n=26)**	0-29	0.00	0.00	111.91	1.23	1.12	1.35	0.00	0.00	125.47	1.0000
	30-49	94.31	30.62	220.10	90.00	88.87	91.15	1.05	0.24	2.90	0.8171
	50-69	313.69	171.50	526.32	283.38	281.13	285.64	1.11	0.51	2.09	0.6714
	70+	434.11	174.53	894.43	324.72	321.52	327.94	1.34	0.41	3.20	0.3792
**Urothelial cancer (n=21)**	0-29	0.00	0.00	55.74	0.26	0.22	0.30	0.00	0.00	301.96	1.0000
	30-49	0.00	0.00	34.66	5.19	5.00	5.39	0.00	0.00	9.19	1.0000
	50-69	82.09	33.00	169.13	61.32	60.57	62.07	1.34	0.41	3.21	0.3786
	70+	496.50	271.44	833.03	169.09	167.30	170.89	2.94	1.34	5.54	**0.0004***
**Prostate cancer (n=19)**	0-29	0.00	0.00	111.04	0.01	0.00	0.02	0.00	0.00	131626.08	1.0000
	30-49	0.00	0.00	69.20	1.57	1.43	1.73	0.00	0.00	60.93	1.0000
	50-69	199.66	86.20	393.40	151.90	150.22	153.59	1.31	0.44	2.99	0.4116
	70+	950.29	474.38	1700.33	621.85	616.44	627.30	1.53	0.62	3.11	0.1852
**Malignant melanoma (n=11)**	0-29	30.26	3.66	109.31	3.28	3.15	3.42	9.22	0.54	41.53	0.0204
	30-49	37.63	10.25	96.34	19.59	19.22	19.97	1.92	0.34	5.90	0.1581
	50-69	23.42	2.84	84.59	34.72	34.16	35.29	0.67	0.04	3.03	1.0000
	70+	106.13	21.89	310.15	47.71	46.76	48.67	2.22	0.27	7.92	0.1543
	0-29	0.00	0.00	55.74	0.25	0.22	0.29	0.00	0.00	306.90	1.0000
**Non-melanoma skin tumours (n=11)**	30-49	9.40	0.24	52.36	2.69	2.56	2.83	3.49	0.02	25.10	0.2492
	50-69	58.67	19.05	136.93	20.79	20.36	21.23	2.82	0.64	7.80	0.0344
	70+	177.04	57.49	413.16	117.75	116.26	119.25	1.50	0.34	4.16	0.3976
**Lung cancer (n=10)**	0-29	0.00	0.00	55.74	0.16	0.14	0.20	0.00	0.00	479.30	1.0000
	30-49	28.19	5.81	82.39	10.92	10.64	11.20	2.58	0.32	9.20	0.1125
	50-69	35.09	7.24	102.55	148.71	147.55	149.88	0.24	0.03	0.84	**0.0028***
	70+	140.43	38.26	359.55	299.16	296.78	301.55	0.47	0.08	1.44	0.1656
**Brain cancer (n=10)**	0-29	15.11	0.38	84.19	5.32	5.16	5.49	2.84	0.02	20.40	0.2968
	30-49	18.80	2.28	67.90	14.80	14.48	15.13	1.27	0.07	5.72	0.6739
	50-69	35.10	7.24	102.57	35.86	35.29	36.44	0.98	0.12	3.48	1.0000
	70+	140.76	38.35	360.39	46.31	45.38	47.26	3.04	0.54	9.33	0.0447
**Non-Hodgkin's lymphoma (n=5)**	0-29	0.00	0.00	55.74	1.44	1.36	1.53	0.00	0.00	53.35	1.0000
	30-49	9.40	0.24	52.39	6.44	6.23	6.66	1.46	0.01	10.48	0.4962
	50-69	35.13	7.25	102.68	25.97	25.49	26.46	1.35	0.17	4.82	0.4909
	70+	35.08	0.89	195.44	56.35	55.32	57.39	0.62	0.00	4.47	1.0000
**Head and neck cancer (n=5)**	0-29	0.00	0.00	55.74	0.30	0.26	0.34	0.00	0.00	256.56	1.0000
	30-49	9.40	0.24	52.36	5.28	5.09	5.47	1.78	0.01	12.79	0.4298
	50-69	23.40	2.83	84.52	29.77	29.25	30.30	0.79	0.05	3.54	1.0000
	70+	70.41	8.53	254.33	36.17	35.35	37.01	1.95	0.11	8.76	0.2744

**Gastric cancer (n=5)**	0-29	0.00	0.00	55.74	0.10	0.08	0.12	0.00	0.00	798.79	1.0000
	30-49	9.40	0.24	52.35	2.68	2.54	2.82	3.51	0.02	25.24	0.2481
	50-69	11.70	0.30	65.16	20.56	20.13	21.00	0.57	0.00	4.08	1.0000
	70+	105.33	21.72	307.81	66.70	65.58	67.83	1.58	0.19	5.62	0.4458
**Ovarian cancer (n=4)**	0-29	0.00	0.00	111.91	1.01	0.91	1.12	0.00	0.00	153.31	1.0000
	30-49	18.82	0.48	104.88	10.66	10.27	11.06	1.77	0.01	12.69	0.4324
	50-69	66.67	13.75	194.83	47.75	46.83	48.68	1.40	0.17	4.97	0.4801
	70+	0.00	0.00	224.31	63.23	61.82	64.66	0.00	0.00	4.88	0.6324
**Endometrial cancer (n=3)**	0-29	0.00	0.00	111.91	0.15	0.12	0.20	0.00	0.00	1052.60	1.0000
	30-49	0.00	0.00	69.43	5.35	5.08	5.64	0.00	0.00	17.89	1.0000
	50-69	66.41	13.70	194.08	62.20	61.15	63.27	1.07	0.13	3.80	0.7623
	70+	0.00	0.00	224.31	78.96	77.39	80.56	0.00	0.00	3.91	0.6456
**Pancreas cancer (n=3)**	0-29	0.00	0.00	55.74	0.04	0.03	0.06	0.00	0.00	2045.06	1.0000
	30-49	0.00	0.00	34.66	2.31	2.19	2.45	0.00	0.00	20.65	1.0000
	50-69	11.69	0.30	65.14	26.24	25.76	26.74	0.45	0.00	3.20	0.7330
	70+	70.19	8.50	253.55	76.49	75.29	77.71	0.92	0.05	4.13	1.0000
**Connecting tissue tumours (n=3)**	0-29	0.00	0.00	55.74	0.82	0.75	0.89	0.00	0.00	94.25	1.0000
	30-49	9.40	0.24	52.35	2.00	1.88	2.12	4.71	0.03	33.89	0.1915
	50-69	23.39	2.83	84.51	4.83	4.62	5.04	4.85	0.28	21.84	0.0650
	70+	0.00	0.00	129.39	9.84	9.41	10.28	0.00	0.00	18.12	1.0000
**Eye tumours (n=3)**	0-29	0.00	0.00	55.74	0.32	0.28	0.37	0.00	0.00	240.15	1.0000
	30-49	0.00	0.00	34.66	0.74	0.67	0.82	0.00	0.00	64.93	1.0000
	50-69	35.11	7.24	102.61	2.54	2.39	2.70	13.81	1.68	49.32	**0.0015***
	70+	0.00	0.00	129.47	4.04	3.77	4.33	0.00	0.00	44.19	1.0000
**Hepatocellular cancer (n=2)**	0-29	0.00	0.00	55.74	0.17	0.14	0.20	0.00	0.00	463.52	1.0000
	30-49	0.00	0.00	34.66	0.98	0.90	1.07	0.00	0.00	48.83	1.0000
	50-69	23.38	2.83	84.47	10.13	9.83	10.44	2.31	0.13	10.39	0.2153
	70+	0.00	0.00	129.43	25.96	25.26	26.67	0.00	0.00	6.86	1.0000
**Laryngeal cancer (n=2)**	0-29	0.00	0.00	55.74	0.02	0.01	0.03	0.00	0.00	4467.07	1.0000
	30-49	0.00	0.00	34.66	1.39	1.30	1.50	0.00	0.00	34.34	1.0000
	50-69	23.40	2.83	84.53	12.63	12.30	12.98	1.85	0.11	8.34	0.2936
	70+	0.00	0.00	129.39	14.47	13.95	15.01	0.00	0.00	12.31	1.0000
**Cervical cancer (n=2)**	0-29	0.00	0.00	111.91	3.25	3.07	3.45	0.00	0.00	47.42	1.0000
	30-49	18.82	0.48	104.87	25.75	25.15	26.37	0.73	0.00	5.25	1.0000
	50-69	22.16	0.56	123.49	26.48	25.79	27.18	0.84	0.01	6.01	1.0000
	70+	0.00	0.00	224.31	28.50	27.56	29.47	0.00	0.00	10.83	1.0000
**Testis cancer (n=2)**	0-29	0.00	0.00	111.04	7.86	7.58	8.15	0.00	0.00	19.45	1.0000
	30-49	37.57	4.55	135.71	19.36	18.85	19.89	1.94	0.11	8.73	0.2756
	50-69	0.00	0.00	91.40	5.48	5.17	5.81	0.00	0.00	22.98	1.0000
	70+	0.00	0.00	305.78	2.80	2.44	3.18	0.00	0.00	152.20	1.0000

**Kidney cancer (n=2)**	0-29	0.00	0.00	55.74	0.48	0.43	0.53	0.00	0.00	160.67	1.0000
	30-49	0.00	0.00	34.66	3.24	3.09	3.40	0.00	0.00	14.72	1.0000
	50-69	11.70	0.30	65.16	21.65	21.21	22.10	0.54	0.00	3.88	1.0000
	70+	35.10	0.89	195.54	43.13	42.23	44.05	0.81	0.01	5.84	1.0000
**Multiple myeloma (n=2)**	0-29	0.00	0.00	55.74	0.01	0.00	0.02	0.00	0.00	19521.01	1.0000
	30-49	0.00	0.00	34.66	1.01	0.93	1.10	0.00	0.00	47.22	1.0000
	50-69	0.00	0.00	43.13	10.20	9.90	10.51	0.00	0.00	5.82	1.0000
	70+	70.29	8.51	253.92	26.70	25.99	27.42	2.63	0.15	11.85	0.1769
**Leukemia (n=1)**	0-29	0.00	0.00	55.74	3.29	3.16	3.42	0.00	0.00	23.36	1.0000
	30-49	0.00	0.00	34.66	4.16	3.99	4.33	0.00	0.00	11.48	1.0000
	50-69	0.00	0.00	43.13	21.29	20.85	21.74	0.00	0.00	2.79	0.2739
	70+	35.13	0.89	195.76	62.52	61.44	63.62	0.56	0.00	4.03	1.0000
**Hodgkin's lymphoma (n=1)**	0-29	15.13	0.38	84.28	2.02	1.92	2.13	7.48	0.05	53.79	0.1252
	30-49	0.00	0.00	34.68	2.52	2.39	2.66	0.00	0.00	18.98	1.0000
	50-69	0.00	0.00	43.13	2.63	2.48	2.79	0.00	0.00	22.62	1.0000
	70+	0.00	0.00	129.39	3.43	3.18	3.70	0.00	0.00	52.08	1.0000
**Esophageal cancer (n=1)**	0-29	0.00	0.00	55.74	0.01	0.00	0.02	0.00	0.00	11813.64	1.0000
	30-49	0.00	0.00	34.66	1.10	1.01	1.19	0.00	0.00	43.56	1.0000
	50-69	11.70	0.30	65.18	13.19	12.84	13.54	0.89	0.01	6.37	1.0000
	70+	0.00	0.00	130.22	27.20	26.49	27.93	0.00	0.00	6.59	1.0000
**Small bowel cancer (n=1)**	0-29	0.00	0.00	55.74	0.03	0.02	0.05	0.00	0.00	2739.32	1.0000
	30-49	0.00	0.00	34.66	0.40	0.35	0.46	0.00	0.00	119.76	1.0000
	50-69	11.70	0.30	65.19	2.86	2.70	3.03	4.09	0.03	29.42	0.2171
	70+	0.00	0.00	129.53	6.32	5.98	6.68	0.00	0.00	28.24	1.0000
**Pleural mesothelioma (n=1)**	0-29	0.00	0.00	55.74	0.01	0.01	0.02	0.00	0.00	8404.69	1.0000
	30-49	0.00	0.00	34.66	0.30	0.26	0.35	0.00	0.00	161.82	1.0000
	50-69	11.69	0.30	65.14	3.47	3.29	3.65	3.37	0.02	24.23	0.2569
	70+	0.00	0.00	129.39	7.17	6.80	7.55	0.00	0.00	24.88	1.0000
**Vaginal and vulva cancer (n=1)**	0-29	0.00	0.00	111.91	0.10	0.07	0.14	0.00	0.00	1648.57	1.0000
	30-49	18.83	0.48	104.92	1.73	1.58	1.90	10.86	0.07	78.43	0.0882
	50-69	0.00	0.00	81.72	6.69	6.35	7.05	0.00	0.00	16.83	1.0000
	70+	0.00	0.00	224.31	21.85	21.02	22.70	0.00	0.00	14.14	1.0000
**Nose and sinuses (n=1)**	0-29	0.00	0.00	55.74	0.06	0.04	0.08	0.00	0.00	1425.04	1.0000
	30-49	0.00	0.00	34.66	0.43	0.38	0.49	0.00	0.00	111.97	1.0000
	50-69	0.00	0.00	43.13	2.39	2.24	2.54	0.00	0.00	24.90	1.0000
	70+	35.17	0.89	195.98	4.76	4.46	5.07	7.39	0.05	53.20	0.1267

*Significant p values following Bonferoni corrections
