# Supplementary material for: Broadening risk profile in familial colorectal cancer type X; increased risk for five cancer types in the national Danish cohort
Source: BMC Cancer. 2020 Apr 22;20:345. doi: 10.1186/s12885-020-06859-5 (PMC7179001; doi:10.1186/s12885-020-06859-5)
Supplement: Supplementary file 5 — Additional file 5 Table S4. Age-dependent incidence rates for cancer types in the entire FCCTX cohort compared to the Danish Lynch syndrome cohort. [file 12885_2020_6859_MOESM5_ESM.pdf]

Supplementary Table 4. Age-dependent incidence rates for cancer types in the entire FCCTX cohort compared to the Danish Lynch syndrome cohort

		FCCTX per 100000 years			Lynch syndrome per 100000 years			FCCTX vs. Lynch syndrome			
**Cancer**	**Age groups**	**IR**	**95% CI lower**	**95% CI Upper**	**IR**	**95% CI lower**	**95% CI Upper**	**IRR**	**95% CI lower**	**95% CI Upper**	**P values**
**Breast cancer**	0-29	0.00	0.00	19.08	9.11	0.23	50.77	0.00	0.00	90.25	0.3621
	30-49	153.79	102.99	220.86	150.70	84.34	248.55	1.02	0.45	2.48	1.0000
	50-69	435.03	326.81	567.62	530.28	349.46	771.53	0.82	0.45	1.55	0.3938
	70+	445.01	271.83	687.29	383.20	104.41	981.13	1.16	0.31	7.16	1.0000
**Prostate cancer**	0-29	0.00	0.00	18.99	0.00	0.00	37.17	NA	0.00	Inf	1.0000
	30-49	0.00	0.00	19.26	0.00	0.00	42.29	NA	0.00	Inf	1.0000
	50-69	210.97	137.81	309.11	391.12	227.84	626.22	0.54	0.24	1.27	0.0549
	70+	742.25	480.35	1095.71	983.36	395.36	2026.09	0.75	0.26	2.77	0.4860
**Urothelial cancer**	0-29	2.58	0.07	14.35	9.57	1.16	34.57	0.27	0.00	11.07	0.2817
	30-49	2.62	0.07	14.59	42.86	18.51	84.46	0.06	0.00	0.65	**0.0009***
	50-69	55.18	30.17	92.59	497.68	365.68	661.81	0.11	0.05	0.24	**<0.0001***
	70+	355.87	238.33	511.09	1130.28	669.87	1786.33	0.31	0.14	0.72	**0.0003***
**Lung cancer**	0-29	0.00	0.00	9.50	0.00	0.00	17.65	NA	0.00	Inf	1.0000
	30-49	13.09	4.25	30.55	37.34	15.01	76.94	0.35	0.06	1.77	0.0707
	50-69	74.75	45.00	116.73	143.60	78.51	240.94	0.52	0.20	1.38	0.0779
	70+	193.14	110.40	313.65	109.68	13.28	396.21	1.76	0.31	33.03	0.7577
**Malignant melanoma**	0-29	7.73	1.59	22.59	14.36	2.96	41.97	0.54	0.04	6.97	0.4283
	30-49	23.59	10.79	44.79	26.80	8.70	62.53	0.88	0.20	4.87	0.7827
	50-69	27.59	11.09	56.84	20.59	2.49	74.38	1.34	0.18	27.76	1.0000
	70+	60.78	19.73	141.83	165.58	34.15	483.90	0.37	0.05	4.07	0.1618
**Non-melanoma skin tumours**	0-29	2.58	0.07	14.36	0.00	0.00	17.65	NA	0.00	Inf	1.0000
	30-49	5.24	0.63	18.92	48.12	22.01	91.35	0.11	0.01	0.73	**0.0012***
	50-69	39.39	18.89	72.44	228.51	143.20	345.96	0.17	0.06	0.46	**<0.0001***
	70+	109.83	50.22	208.49	462.53	199.69	911.38	0.24	0.06	0.94	**0.0046***
**Brain tumours**	0-29	7.73	1.59	22.60	9.57	1.16	34.57	0.81	0.05	20.46	1.0000
	30-49	13.10	4.25	30.57	53.46	25.63	98.31	0.25	0.05	1.04	**0.0102***
	50-69	31.47	13.59	62.01	92.53	42.31	175.66	0.34	0.09	1.31	0.0283
	70+	72.50	26.61	157.80	0.00	0.00	204.83	NA	0.16	Inf	0.5993
**Pancreatic cancer**	0-29	0.00	0.00	9.51	0.00	0.00	17.65	NA	0.00	Inf	1.0000
	30-49	0.00	0.00	9.66	5.34	0.14	29.73	0.00	0.00	78.01	0.3291
	50-69	27.53	11.07	56.72	71.76	28.85	147.86	0.38	0.08	1.76	0.0746
	70+	169.16	92.48	283.82	274.66	89.18	640.95	0.66	0.18	3.35	0.3872
**Gastric cancer**	0-29	0.00	0.00	9.50	0.00	0.00	17.65	NA	0.00	Inf	1.0000
	30-49	15.71	5.76	34.19	21.36	5.82	54.68	0.74	0.12	5.51	0.7383
	50-69	15.73	4.29	40.27	123.79	63.97	216.24	0.13	0.02	0.55	**0.0001***
	70+	120.69	57.88	221.96	275.53	89.47	643.00	0.44	0.10	2.38	0.1672

**Ovarian cancer**	0-29	10.31	1.25	37.26	0.00	0.00	33.61	NA	0.05	Inf	0.5384
	30-49	10.51	1.27	37.95	252.67	163.51	372.99	0.04	0.00	0.22	**<0.0001***
	50-69	69.27	31.67	131.49	96.36	31.29	224.86	0.72	0.16	3.98	0.5594
	70+	84.24	22.95	215.70	0.00	0.00	343.62	NA	0.09	Inf	1.0000
**Non-Hodgkin's lymphoma**	0-29	0.00	0.00	9.50	4.78	0.12	26.66	0.00	0.00	85.61	0.3500
	30-49	7.86	1.62	22.96	5.33	0.14	29.72	1.47	0.07	313.00	1.0000
	50-69	31.52	13.61	62.10	0.00	0.00	37.78	NA	0.43	Inf	0.1164
	70+	48.36	13.18	123.81	0.00	0.00	202.26	NA	0.09	Inf	1.0000
**Head and neck tumours**	0-29	0.00	0.00	9.50	0.00	0.00	17.65	NA	0.00	Inf	1.0000
	30-49	5.24	0.63	18.91	0.00	0.00	19.68	NA	0.04	Inf	1.0000
	50-69	35.43	16.20	67.25	10.24	0.26	57.07	3.46	0.34	612.79	0.3020
	70+	48.40	13.19	123.92	55.34	1.40	308.34	0.87	0.05	174.25	1.0000
**Endometrial cancer**	0-29	0.00	0.00	19.02	0.00	0.00	33.61	NA	0.00	Inf	1.0000
	30-49	0.00	0.00	19.37	688.64	535.80	871.52	0.00	0.00	0.04	**<0.0001***
	50-69	53.71	21.59	110.65	1686.10	1355.83	2072.51	0.03	0.01	0.08	**<0.0001***
	70+	83.83	22.84	214.63	367.68	100.18	941.39	0.23	0.03	1.92	0.0441
**Kidney cancer**	0-29	0.00	0.00	9.50	0.00	0.00	17.65	NA	0.00	Inf	1.0000
	30-49	2.62	0.07	14.59	5.33	0.14	29.72	0.49	0.00	156.30	0.5500
	50-69	23.59	8.66	51.35	102.83	49.31	189.11	0.23	0.05	0.91	**0.0038***
	70+	36.18	7.46	105.75	110.52	13.38	399.25	0.33	0.02	8.29	0.2207
**Cervical cancer**	0-29	0.00	0.00	19.02	9.11	0.23	50.76	0.00	0.00	89.99	0.3614
	30-49	26.34	8.55	61.47	70.21	28.23	144.66	0.38	0.06	1.90	0.1242
	50-69	23.15	4.77	67.64	38.07	4.61	137.51	0.61	0.04	15.40	0.6303
	70+	21.01	0.53	117.05	0.00	0.00	342.71	NA	0.00	Inf	1.0000
**Leukemia**	0-29	0.00	0.00	9.50	0.00	0.00	17.65	NA	0.00	Inf	1.0000
	30-49	0.00	0.00	9.66	0.00	0.00	19.68	NA	0.00	Inf	1.0000
	50-69	11.79	2.43	34.45	10.24	0.26	57.08	1.15	0.05	244.51	1.0000
	70+	48.32	13.16	123.71	54.91	1.39	305.96	0.88	0.05	175.31	1.0000
**Esophageal cancer**	0-29	0.00	0.00	9.50	0.00	0.00	17.65	NA	0.00	Inf	1.0000
	30-49	0.00	0.00	9.66	5.33	0.14	29.72	0.00	0.00	78.04	0.3292
	50-69	15.72	4.28	40.25	30.74	6.34	89.85	0.51	0.05	6.02	0.4049
	70+	24.17	2.93	87.33	0.00	0.00	202.26	NA	0.02	Inf	1.0000
**Connecting tissue tumours**	0-29	0.00	0.00	9.50	4.79	0.12	26.67	0.00	0.00	85.58	0.3499
	30-49	5.24	0.63	18.92	5.34	0.14	29.74	0.98	0.02	234.47	1.0000
	50-69	11.80	2.43	34.47	30.76	6.34	89.89	0.38	0.03	4.97	0.3573
	70+	12.06	0.31	67.18	55.57	1.41	309.63	0.22	0.00	69.11	0.3248
**Testis cancer**	0-29	5.15	0.13	28.69	30.28	6.24	88.48	0.17	0.00	3.75	0.1152
	30-49	26.21	8.51	61.17	34.59	7.13	101.09	0.76	0.10	8.39	0.7113
	50-69	0.00	0.00	29.77	0.00	0.00	84.04	NA	0.00	Inf	1.0000
	70+	0.00	0.00	104.73	0.00	0.00	501.29	NA	0.00	Inf	1.0000

**Biliary tract cancer**	0-29	0.00	0.00	9.50	0.00	0.00	17.65	NA	0.00	Inf	1.0000
	30-49	5.24	0.63	18.91	5.33	0.14	29.72	0.98	0.02	234.61	1.0000
	50-69	7.86	0.95	28.39	10.24	0.26	57.07	0.77	0.02	183.36	1.0000
	70+	12.06	0.31	67.18	54.83	1.39	305.50	0.22	0.00	70.03	0.3280
**Eye tumours**	0-29	0.00	0.00	9.50	0.00	0.00	17.65	NA	0.00	Inf	1.0000
	30-49	0.00	0.00	9.66	10.68	1.29	38.57	0.00	0.00	5.71	0.1082
	50-69	19.66	6.38	45.88	10.25	0.26	57.11	1.92	0.14	366.93	1.0000
	70+	0.00	0.00	44.53	0.00	0.00	202.26	NA	0.00	Inf	1.0000
**Multiple myeloma**	0-29	0.00	0.00	9.50	0.00	0.00	17.65	NA	0.00	Inf	1.0000
	30-49	0.00	0.00	9.66	5.33	0.14	29.72	0.00	0.00	78.02	0.3292
	50-69	3.93	0.10	21.89	10.25	0.26	57.11	0.38	0.00	122.07	0.4774
	70+	48.28	13.16	123.62	109.67	13.28	396.18	0.44	0.04	10.26	0.2966
**Laryngeal cancer**	0-29	0.00	0.00	9.50	0.00	0.00	17.65	NA	0.00	Inf	1.0000
	30-49	0.00	0.00	9.66	0.00	0.00	19.68	NA	0.00	Inf	1.0000
	50-69	15.73	4.28	40.27	30.79	6.35	89.99	0.51	0.05	6.02	0.4047
	70+	12.08	0.31	67.32	0.00	0.00	202.67	NA	0.00	Inf	1.0000
**Hepatocellular cancer**	0-29	0.00	0.00	9.50	0.00	0.00	17.65	NA	0.00	Inf	1.0000
	30-49	0.00	0.00	9.66	5.33	0.14	29.72	0.00	0.00	78.02	0.3292
	50-69	15.72	4.28	40.24	30.76	6.34	89.89	0.51	0.05	6.02	0.4048
	70+	0.00	0.00	44.48	109.67	13.28	396.16	0.00	0.00	2.56	0.0325
**Small bowel cancer**	0-29	0.00	0.00	9.50	0.00	0.00	17.65	NA	0.00	Inf	1.0000
	30-49	2.62	0.07	14.59	42.73	18.45	84.20	0.06	0.00	0.66	**0.0009***
	50-69	3.93	0.10	21.90	93.04	42.54	176.62	0.04	0.00	0.43	**0.0001***
	70+	12.06	0.31	67.20	110.95	13.44	400.80	0.11	0.00	4.47	0.0843
**Vaginal and vulva cancer**	0-29	0.00	0.00	19.02	0.00	0.00	33.61	NA	0.00	Inf	1.0000
	30-49	5.25	0.13	29.25	0.00	0.00	36.79	NA	0.00	Inf	1.0000
	50-69	7.67	0.19	42.76	37.44	4.53	135.25	0.20	0.00	8.43	0.2045
	70+	20.96	0.53	116.79	0.00	0.00	339.29	NA	0.00	Inf	1.0000
**Thyroid cancer**	0-29	0.00	0.00	9.50	0.00	0.00	17.65	NA	0.00	Inf	1.0000
	30-49	0.00	0.00	9.66	5.33	0.14	29.72	0.00	0.00	78.03	0.3292
	50-69	3.93	0.10	21.90	10.25	0.26	57.12	0.38	0.00	122.09	0.4774
	70+	12.07	0.31	67.24	54.84	1.39	305.55	0.22	0.00	70.09	0.3282
**Pleural mesothelioma**	0-29	0.00	0.00	9.50	0.00	0.00	17.65	NA	0.00	Inf	1.0000
	30-49	0.00	0.00	9.66	0.00	0.00	19.68	NA	0.00	Inf	1.0000
	50-69	3.93	0.10	21.89	0.00	0.00	37.78	NA	0.00	Inf	1.0000
	70+	0.00	0.00	44.48	54.84	1.39	305.55	0.00	0.00	34.96	0.1802
**Bone cancer/osteosarcoma**	0-29	2.58	0.07	14.35	4.79	0.12	26.68	0.54	0.00	171.33	1.0000
	30-49	0.00	0.00	9.66	0.00	0.00	19.68	NA	0.00	Inf	1.0000
	50-69	0.00	0.00	14.49	0.00	0.00	37.78	NA	0.00	Inf	1.0000
	70+	0.00	0.00	44.48	0.00	0.00	202.26	NA	0.00	Inf	1.0000

**Hodgkin's lymphoma**	0-29	2.58	0.07	14.36	0.00	0.00	17.65	NA	0.00	Inf	1.0000
	30-49	0.00	0.00	9.66	0.00	0.00	19.68	NA	0.00	Inf	1.0000
	50-69	0.00	0.00	14.49	10.24	0.26	57.07	0.00	0.00	60.99	0.2772
	70+	0.00	0.00	44.48	0.00	0.00	202.26	NA	0.00	Inf	1.0000
**Nose and sinuses**	0-29	0.00	0.00	55.74	0.00	0.00		NA	0.00	Inf	1.0000
	30-49	0.00	0.00	34.66	0.00	0.00		NA	0.00	Inf	1.0000
	50-69	0.00	0.00	43.13	0.00	0.00		NA	0.00	Inf	1.0000
	70+	35.17	0.89	195.98	0.00	0.00		NA	0.00	Inf	1.0000

*Significant p values following Bonferoni corrections
